# Financial capability, health and disability

**DOI:** 10.1186/s12889-015-1589-5

**Published:** 2015-03-14

**Authors:** Peter Allmark, Katarzyna Machaczek

**Affiliations:** Centre for Health and Social Care Research, Sheffield Hallam University, Sheffield, UK

**Keywords:** Financial, Capability, Approach, Sen, Nussbaum, Disability, Inequality, Wellbeing, Social Prescribing

## Abstract

**Background:**

It has been suggested that improving people’s ability to deal with their finances, their financial capability, will directly improve their wellbeing and indirectly their health. To this end, financial capability initiatives have been funded by statutory and charitable health bodies, sometimes as part of a practice termed ‘social prescribing’.

**Discussion:**

This paper examines financial capability from the perspective of the Capability Approach to welfare and justice. It argues that the Approach shows current conceptions of financial capability to be flawed in that they focus on it as a personal quality in isolation from the socioeconomic environment. Using the Capability Approach as applied to disability the paper argues that financial capability is best viewed as a ‘conversion factor’ rather than a capability, that is, something necessary to convert resources, particularly money, into something of value to an individual, such as an adequate pension. Often, those judged as lacking financial capability are poor and this fact is at the heart of their inability to, say, plan a pension; by contrast, those who are not poor may find it relatively easy to do so and thus be deemed financially capable. Hence there are two distinct types of financial capability: i) in poverty and ii) not in poverty. To be able to plan a pension or make ends meet in poverty requires distinct and perhaps rare skills in an individual. However, some environmental or social changes may help individuals to improve their financial capability without calling on them to develop extraordinary abilities. Given the potential of such work to improve people’s health, making such changes can reasonably be described as Public Health work. The article concludes with a defence of this use of the Capability Approach against possible criticism.

**Summary:**

The Capability Approach enables analysis of financial capability that is theoretically important to and has practical implications for Public Health.

## Background

Approximately, financial capability is people’s ability to deal with their finances by such things as planning their pension and making ends meet. A shortfall in people’s financial capability has been identified as partially to blame for bad financial planning, such as a lack of personal pension provision [1]. Raising the national level of financial capability, particularly amongst the poorest in the population, has become a target of a number of Western Governments. In the UK, for example, State and Third-Sector bodies have been charged with the task of improving financial capability through, for example, financial education in schools [2-4]. One potential benefit of this would be improvement in wellbeing, particularly psychological; a review of data from the British Household Panel Survey from 1991–2006 found a strong association between financial capability and psychological wellbeing [5]. They conclude that these findings are ‘consistent with the hypothesis that changes in financial capability lead to changes in psychological wellbeing’ ([5]; p.5). There is also evidence that related features of financial capability, such as avoidance of debt, have health benefits [6,7]. The mechanism for such benefits is not straightforward; the Joseph Rowntree Foundation recently published a review of 272 papers setting out theories examining how income affects health. Amongst these were empirical studies showing how the stress of managing low income can lead to biochemical changes that cause ill health ([8]: especially pp. 35–38). Insofar as financial capability work reduces such stress, an inverse effect might be expected.

Because of such considerations, financial capability initiatives have been viewed as potential public health initiatives and perhaps worth funding as such through, for example, the National Health Service [6]. This has sometimes been done as part of a practice termed ‘social prescribing’. If successful, initiatives to enhance financial capability would have the side-benefit of reducing health inequality between rich and poor as well as improving the health of those targeted. Up to now, these interventions have primarily been education and the evidence for the effectiveness of financial capability training is weak [5], a point we return to later.

This paper will draw upon the Capability Approach to assessing welfare and social justice to argue that financial capability as presently conceptualised is flawed. It will suggest that the idea of improving people’s ability to deal with their financial situation, and indirectly to improve health, can be rescued from the problems but that doing so has practical implications. We start with a commonly used definition of financial capability.

## Discussion

### World Bank definition of financial capability

In a 2013 report *Making Sense of Financial Capability Surveys around the World,* The World Bank draws together the notions of financial wellbeing, financial capability and financial literacy [9]. Financial wellbeing exists where individuals get the financial services to fit their needs, such as adequate pension planning. Financial literacy is people’s knowledge and awareness of financial concepts and products. Financial literacy is not enough on its own to ensure Financial Wellbeing; the individual will also need the absence of internal barriers to behaving in her best financial interest. For example, someone with drug addiction problems may still fall into debt or destitution despite financial literacy. Thus the Report’s final definition of Financial Capability is:

‘the internal capacity to act in one’s best financial interest given socioeconomic environmental conditions. It therefore encompasses the knowledge, attitudes, skills and behaviors of consumers with regard to managing their resources and understanding, selecting and making use of financial services that fit their needs’ (p. 7).

### The capability approach

The use of the term ‘capability’ leads some to posit a link to the Capability Approach to social justice developed Armatya Sen and Martha Nussbaum [10,11]. At the core of the Capability Approach is the concept of people’s functionings, what they do and are. Examples of ‘doing’ are travelling, going to work, washing and reading. Examples of ‘what you are’ (or ‘beings’) are educated, healthy, isolated and undernourished. People’s wellbeing is grounded in these functionings; and the functionings emerge from their choices. Capabilities are the free choices we have; functionings the results of the choices we make. Individuals’ conceptions of a good life, of wellbeing, differ; the steam trains adored by one person will bore another. Hence although watching steam trains is an option for many people, few will do it: watching steam trains is a capability for many but a functioning for few.

Our choices are constrained in various ways: some are impossible for all; we cannot choose to fly unaided. Some are impossible for many; most cannot buy a Lamborghini. Some are possible but only with mighty sacrifice; wealthier people might be able to buy the Lamborghini but only by selling their house. Thus we may think of our choices as belonging in sets. I might have a set which contains a house and another which contains a Lamborghini but I do not have a set which contains both. Other people’s sets are restricted in more important ways; for example, they may be able to earn enough to be adequately nourished but only by working so many hours they barely spend time with their family. These discrete packages of choices are our Capability sets. We might think of our complete set of capability sets as our overall Capability, that is, what we can be and do.

The overall Capability of each individual is a product of the resources available to people plus their ability to convert the resource into a functioning, such as the ability to convert a bicycle into a means of transport or wages into adequate pension provision. Some authors denote this ability a ‘conversion factor’ [12,13]. Conversion factors have three elements: personal, environmental and social. Thus in order for a woman to convert food into adequate nutrition she needs to be: i) Personally well enough to absorb the nutrition; ii) Environmentally placed such that she can obtain the food without, say, having to cross a dangerous desert; and iii) In a social position where such things as norms, laws and power relations permit her to obtain and eat the food.

**Figure 1:**
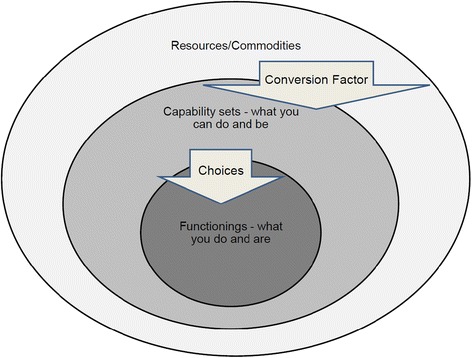
**The Capability Approach - based on Robeyns** **[** 12 **]** **and Marron** **[** 13 **]** **.**

The Capability Approach is summarised in Figure 1.

In order for the Capability Approach to be used to judge the quality of life and justice of social arrangements we need to identify what choices, or capabilities, are required for someone to have adequate life quality. If I feel my life is rendered imperfect or unhappy by my inability to own a Lamborghini then it would seem reasonable for others to criticise me rather than social arrangements, pointing to my family, home and various other circumstances as reasons that I should be content. By contrast, the people unable to spend time with their families because they have to work so many hours to get money for food would seem to have a *prima facie* cause to complain of injustice.

To show why the person lacking family time has cause for complaint (whilst the one lacking a Lamborghini does not) we can turn to Nussbaum. She suggests ten core capabilities all of which need to be present within at least one of a person’s capability sets if that person is to have a chance of a satisfactory life quality [10]: these relate to life; bodily health; bodily integrity; senses, imagination and thought; emotions; practical reason; affiliation; other species; play; and control over one’s political and material environment. The precise details of these need not concern us but we should note that Nussbaum views these as ends rather than means; in order to live well individuals must have the freedom to, say, take part in political life or enjoy a life of reasonable length and health.

### Financial capability and the capability approach

It follows that financial capability is not a capability of the Sen/Nussbaum type. For most people it is a means to ends that are genuinely constitutive of a good life, such as adequate nutrition and health, control and the ability to play. Financial capability might however be considered a conversion factor necessary for an individual to convert a resource, money, into various financial goods. In modern Western societies this would include, for example, adequate pension planning either alone or with state help. Financial capability as a conversion factor is people’s ability to convert their resources (the outer ring in Figure 1) to capability sets that include the necessary financial goods (the middle ring). Whether or not the individual chooses to make the conversion is down to them; if they do, it will be part of their functionings.

If financial capability is best conceived of as a conversion factor, is lack of financial capability equivalent to a kind of disability, of the type discussed extensively by Nussbaum [10]? Let us return to the example of the woman and her ability to convert wages into adequate nutrition. Extend this to two women receiving the same income. One, call her Margaret, has no major health problems; the other, Josie, is diabetic and wheelchair-bound. Margaret finds getting adequate nutrition straightforward; she is able to get to the supermarket, find the food she needs, buy cheap offers and so on. She is also left with enough money to fund a reasonable social life and to plan her future finance. Josie struggles to get by on the same money: she has to pay carers to help her go shopping and has to buy special foods, which are expensive. The carers used to be provided by the local council but this has recently been cut. Although she manages to feed herself, she has to sacrifice things she values, such as trips out, and her pension plan is inadequate. We might say that her overall Capability is far more constrained than Margaret’s. In this example, Josie’s lack of a pension and of money for trips out might be described as her lacking the conversion factor to do so. But that lack is not just of a personal physical element, her wheelchair-dependence and diabetes. It also has social and environmental elements, such as the lack of an enabling transport system and lack of welfare provision. As Nussbaum puts it,

“To realize one of the items on the list [of core capabilities] … entails not only promoting appropriate development of their internal powers, but also preparing the environment so that it is favourable for the exercise of practical reason and the other major functions.” ([14]; p.85)

Nussbaum acknowledges that in some cases personal disability puts a core functioning out of someone’s reach; someone with severe mental handicap might lack the ability to take part in political life no matter how enabling the social arrangements. But in general, we should consider conversion factors as a personal, social and environmental package.

Taking financial capability to be a type of disability exposes a problem with its usual formulation. A conversion factor has social, environmental and individual elements. A disability indicates that the individual element is such that adjustments need to be made to the social and environmental elements in order for the person to convert resources to capability sets. If we take tetraplegia as an example of disability, in order to achieve mobility such an individual requires additional resources from society (to pay for a wheelchair, say) plus changes in the environment, such as wheelchair ramps. However, this is not the case where someone is said to lack financial capability. Such an individual generally has fewer financial resources to begin with and thus requires additional individual skills in order to achieve the endpoint of adequate financial planning over a lifespan. Such people are not being required to plan an adequate pension but rather to plan an adequate pension from a position of poverty. In this sense, the financial capability of someone in poverty is a different feature from the financial capability of someone not. It would be better therefore to talk of the conversion factor as ‘financial capability in poverty’ to remove the ambiguity. Much of the charitable and public sector work is thus about developing ‘financial capability in poverty’.

### Health and ‘financial capability in poverty’

We have argued that financial capability is not a capability in the sense of the Capability Approach, but this is not in itself a flaw; those using the term financial capability do not need to make such a claim. The flaw lies in the judgement of financial capability or incapability being made without adequate heed of the starting points of the individuals so judged. This can be illustrated using the model of the Capability Approach presented in Figure 1. This shows three nested circles: resources/commodities; capability sets; and functionings. Resources and capability sets are linked by Conversion Factors; capability sets and functionings are linked by choices. The main difference between those in poverty and those not is the size of the outer circle; poverty means fewer resources. It follows that some different conversion factors may be required for those with limited resources to achieve important functionings; ‘financial capability in poverty’ is one such conversion factor.

What are the implications for those working in Public Health? The motivation to work with financial capability arises from observation of a link between health and finance, as set out above. As such it is reasonable to consider work to improve financial capability of those in poverty as work for Public Health. Once financial capability is reconceived as ‘financial capability in poverty’ a problem with the approach becomes apparent; would it not be better not to have the poverty in the first place? This may be so but it is surely beyond the remit of Public Health to make the necessary changes, even if we were agreed what those are.

Given that there is poverty, would not the development of ‘financial capability in poverty’ be reasonable Public Health work? The answer is in the affirmative but with the caveat that this capability is seen from the Capability Approach perspective as a conversion factor, with individual, social and environmental elements. Nussbaum’s writings provide many examples of people who manage in poverty to live well; but they often require extraordinary skills to do so; only with such skills will they have the conversion factor to turn their resources over a lifetime into education, food, health and so on.

Thus one problem with financial capability work when conceived as correcting shortfalls in the abilities of individuals is that it might be trying to develop the extraordinary in people who are for the most part ordinary. It might simply be unreasonable to expect people in poverty to live life free of short-term high-interest debt, for example. Furthermore, the environment might be such as to create coping strategies that are undesirable, such as benefit fraud, or unsustainable, such as the building of multiple loans to offset each other. However, once financial capability work is conceived of more widely, to include environmental and social factors, there are social and environmental changes that professionals and volunteers can make at various levels. Examples include: making cheaper finance available through Credit Unions; working with the Police to remove loan sharks; helping individuals with debt; removing barriers to employment; and ensuring individuals take benefits and tax breaks they are due.

Often individuals in poverty will face a network of problems, some of which might be related to individual shortfalls in ability or education. As such, there may be skills that can be developed that help individuals cope with their limited finances. It is plausible that individuals will have shortfalls in ability or education that can be offset through, for example, training; but this needs to be set against the point already made that we should not expect ordinary people to develop extraordinary abilities.

It is not clear whether such work is best described as developing ‘financial capability in poverty’ (or financial capability work) or simply as helping individuals out of poverty, but perhaps not too much rests on this distinction. It might also be said that developing ‘financial capability in poverty’ is not rightly conceived of as Public Health work any more than is work in other areas of social policy such as education; just as work to improving ‘financial capability in poverty’ improves health so does improving educational attainment. Again, it may be that not too much rests on the distinction; the key point is that financial capability work, properly conceived as a social, environmental and individual package, has potential to improve the public’s health and to reduce health inequality - both of which are goals of Public Health. Whether it is described as Public Health or not is probably a function of the intention with which it is undertaken; our belief is that it could be undertaken with a direct Public Health intention. At present, the concept as described by the WHO or the Financial Services Authority is developed on the basis of outcomes such as making ends meet or planning an adequate pension. This and with it the current approach to enhancing financial capability over-emphasizes the individual element of the package.

## Summary

This article has put the Capability Approach to the analysis of Public Health work that aims to improve health through improving financial capability. The conclusions are both theoretical and practical. On the theory side, financial capability is better characterised i) as a conversion factor rather than a capability and ii) as a factor that is different in different circumstances, particularly in poverty or not. On the practice side, rather than say that the poor unlike others lack an ability to make ends meet we should recognise that making ends meet in poverty requires particular and perhaps rare abilities and emphasise instead the environmental and social elements of the conversion factor.

Someone might argue that the Capability Approach has added little here other than a layer of unnecessary theoretical complexity; practitioners already do the combination of social, environmental and individual work for health in poverty; their basis for this is either some kind of intuition or common-sense, or, perhaps, a more straightforward and widely used theory such as those based on utilitarian style calculations (such as welfarism) [15]. Tackling this criticism in full would require a second article. However, we would make the following observation.

If there were intuitive (or other) agreement on how financial capability is achieved and how it is related to health then there would be no need to examine it analytically as we have here. However, there is not. There is evidence that financial capability is wrongly conceived of as primarily an individual attribute, notably lacking in the poor. Alongside, there is disagreement about how to develop it and a notable lack of success in doing so. The analysis in this article points to why this might be so.
